# Evaluation of New Approaches to Depression Treatment Using an Animal Model of Pharmacoresistant Depression

**DOI:** 10.3390/ijms25105265

**Published:** 2024-05-12

**Authors:** Alexandra Zvozilova, Stanislava Bukatova, Romana Koprdova, Mojmir Mach

**Affiliations:** 1 Centre of Experimental Medicine of the Slovak Academy of Sciences, Institute of Experimental Pharmacology and Toxicology, 841 04 Bratislava, Slovakia; exfaalba@savba.sk (A.Z.); stanislava.bukatova@savba.sk (S.B.); romana.koprdova@savba.sk (R.K.); 2 Department of Pharmacology, Jessenius Faculty of Medicine in Martin, Comenius University in Bratislava, 036 01 Martin, Slovakia

**Keywords:** treatment-resistant depression, antidepressants, Wistar-Kyoto rats, behavior, pyridoindoles, zoletil, venlafaxine

## Abstract

Depression is emerging as the predominant psychiatric disorder globally. Despite the wide availability of antidepressants, up to 30% of patients exhibit poor response to treatment, falling into the category of treatment-resistant depression (TRD). This underscores the need for the exploration of novel therapeutic options. Our work aims to study the effect of chronic administration of the pyridoindole derivative SMe1EC2M3, a triple reuptake inhibitor, and the combination of zoletil and venlafaxine under conditions of stress induced by a 4-week chronic mild stress (CMS) procedure in Wistar-Kyoto male rats as an animal model of TRD. Therefore, we investigated the possible effect of the selected compounds in four experimental groups, i.e., stress + vehicle, stress + venlafaxine, stress + zoletil + venlafaxine and stress + SMe1EC2M3. The following variables were assessed: anhedonia in sucrose preference test (SPT), spontaneous locomotion and exploration in open field test (OF), anxiety-like behavior in elevated plus maze test (EPM), motivation and depressive-like behavior in forced swim test (FST) and nociception in tail flick test. We also evaluated cognition, particularly recognition memory, in the novel object recognition test (NOR). Sucrose preference was significantly increased in the SMe1EC2M3 group (*p* < 0.05) in comparison with the venlafaxine animals. In the OF, we observed a significantly higher number of entries into both the central and peripheral zones in the venlafaxine (*p* < 0.05 central zone; *p* ≤ 0.05 periphery zone) and SMe1EC2M3 (*p* < 0.05 central zone; *p* < 0.05 periphery zone) groups compared to the venlafaxine + zoletil group. SMe1EC2M3 was able to significantly increase the time of climbing in FST (*p* < 0.05) in comparison with the venlafaxine and control groups. The NOR test revealed a significantly higher discrimination ratio in the SMe1EC2M3 group (*p* < 0.05) compared to the control and venlafaxine groups. Analyses of the tail flick test showed a significant increase in reaction time to painful stimuli in the SMe1EC2M3 group (*p* < 0.05) in comparison to both the control and venlafaxine groups. Our findings suggest that SMe1EC2M3 has the potential to ameliorate some behavioral changes associated with TRD, and the venlafaxine + zoletil combination treatment was not a promising treatment alternative in the animal model of TRD.

## 1. Introduction

Major Depressive Disorder (MDD) is a multifactorial mental disease which is one of the leading causes of disability globally [1,2]. The exact etiology of MDD is still not clearly understood. The well-known monoamine theory of depression has support from the mechanism of action of antidepressants, which modulate monoaminergic systems [3]. Nowadays, the first-line antidepressants are a group of selective serotonin (5-HT) reuptake inhibitors (SSRIs) such as fluoxetine, followed by serotonin and noradrenaline (NE) reuptake inhibitors such as venlafaxine. They effectively increase the availability of these monoamines in the synaptic cleft [4]. Antidepressants from the group of triple reuptake inhibitors (TRIs) have a unique ability to increase the availability of 5-HT, NE and DA, making them promising candidates for effective treatment of MDD [5]. Studies are showing that simultaneous modulation of all three monoamine systems heightens their clinical efficiency in treatment [6,7].

Clinical evidence shows that nearly 60% of patients do not achieve a sufficient response after the first antidepressant trial of an adequate dose and duration. Furthermore, approximately 30% of MDD patients do not respond to any commercially available drugs, falling into the category of treatment-resistant depression (TRD) [6,8,9]. TRD does not have a precise definition; however, it can be considered when a patient does not respond to first-line antidepressant treatment, typically SSRIs, and to subsequent treatment with another class of antidepressants with a different mechanism of action [10]. Despite numerous studies, the origin of and factors influencing the development of TRD remain unknown. Internal physiological factors and environmental genetic heterogeneity among patients might be responsible for unsuccessful recovery and symptom remission in TRD [11]. Studies suggest that the genes expressed in patients with TRD are primarily associated with glutamatergic and monoaminergic neurotransmission, as well as synaptic plasticity. This hypothesis is highly supported by the effective treatment with the non-competitive N-methyl-D-aspartate (NMDA) glutamate receptor antagonist ketamine as a rapidly acting anesthetic [12,13]. Ketamine can increase the availability of glutamate, leading to increased synaptogenesis and elevated concentrations of brain-derived neurotrophic factor (BDNF) [14]. Experimental studies on rodents using ketamine in the treatment of TRD indicate a diminishment of some depressive-like symptoms [13,15]. However, the limitation of ketamine use is its addictive properties and the authors in the study administered ketamine for only one week. Based on these studies, a veterinary anesthetic zoletil, as a combination of tiletamine and zolazepam, might offer similar effects in the treatment of TRD to ketamine. Tiletamine is an NMDA antagonist and zolazepam is an effective benzodiazepine with a much higher antianxiogenic potential than diazepam [16,17]. The aim of this paradigm was to explore zoletil’s “boosting” potential for commonly administered antidepressants, which could be then more effective in the treatment of TRD.

**Figure 1:**
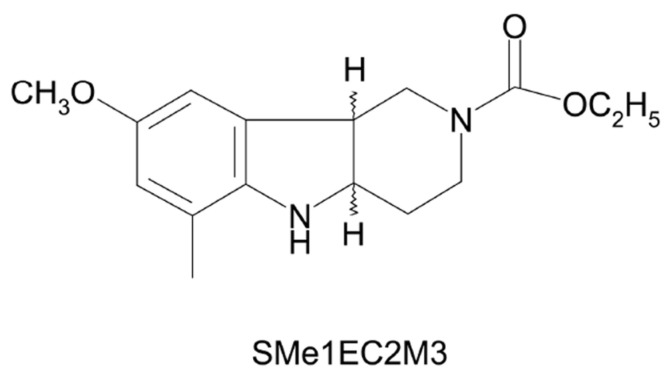
Structural formula of (±)-*cis* ethyl 8-methoxy-6-methyl-3,4,4a,5,9b*H*-hexahydro-1*H*pyrido [4,3-b] indole-2-carboxylate (SMe1EC2M3).

Based on our previous research, a promising option in the treatment of TRD might include a triple reuptake inhibitor: the pyridoindole derivative (±)-*cis* ethyl 8-methoxy-6-methyl-3,4,4a,5,9b*H*-hexahydro-1*H*-pyrido [4,3-b] indole-2-carboxylate (SMe1EC2M3, Figure 1). It was synthesized at the Institute of Experimental Pharmacology and Toxicology, Slovak Academy of Sciences, and originally developed for treatment after brain injuries with antioxidant activity [18]. SMe1EC2M3 increases c-Fos-positive cells after acute administration, particularly in regions associated with the pathogenesis of depression, such as the central amygdaloid nucleus and nucleus paraventricularis of the hypothalamus [19]. The results of in vivo electrophysiology suggested that SMe1EC2M3 might act as a 5-HT, NE and DA reuptake inhibitor, supported by a significant reduction in immobility and an increase in active swimming during the forced swim test [19,20]. Our recent study showed that the derivative has no neuronal cytotoxic side effects and has antidepressant properties after chronic administration, and might ameliorate effect of chronic mild stress (CMS) in gliogenesis and the proliferation of progenitors in hippocampal dentate gyrus [21].

Studies on animal models of TRD suggest using Wistar-Kyoto (WKY) rats, which were originally bred as a normotensive control for spontaneously hypertensive rat lines. Research revealed physiological and behavioral characteristic changes of increased emotionality [22]. It is known that this line did not respond to treatment with first- and second-line antidepressants, such as the tricyclic antidepressant imipramine, the SSRI fluoxetine, or the SNRI antidepressant venlafaxine [13,23,24]. It correlates with behavioral tests such as the sucrose preference test (SPT), the elevated plus maze test (EPM), and the novel object recognition test (NOR), where the treatment did not ameliorate symptoms of depression-like or anxiety-like behavior. The administration of ketamine after eight days resulted in a complete reversal of symptoms. The model of CMS applied to the WKY rat line can be considered a valid animal model of TRD [13,25,26].

The aim of the current study was to further explore the antidepressant effects of SMe1EC2M3 in our animal model of TRD using Wistar-Kyoto male rats under conditions of CMS as well as to test an alternative treatment strategy for TRD by combination therapy with zoletil and venlafaxine.

## 2. Results

**Figure 2:**
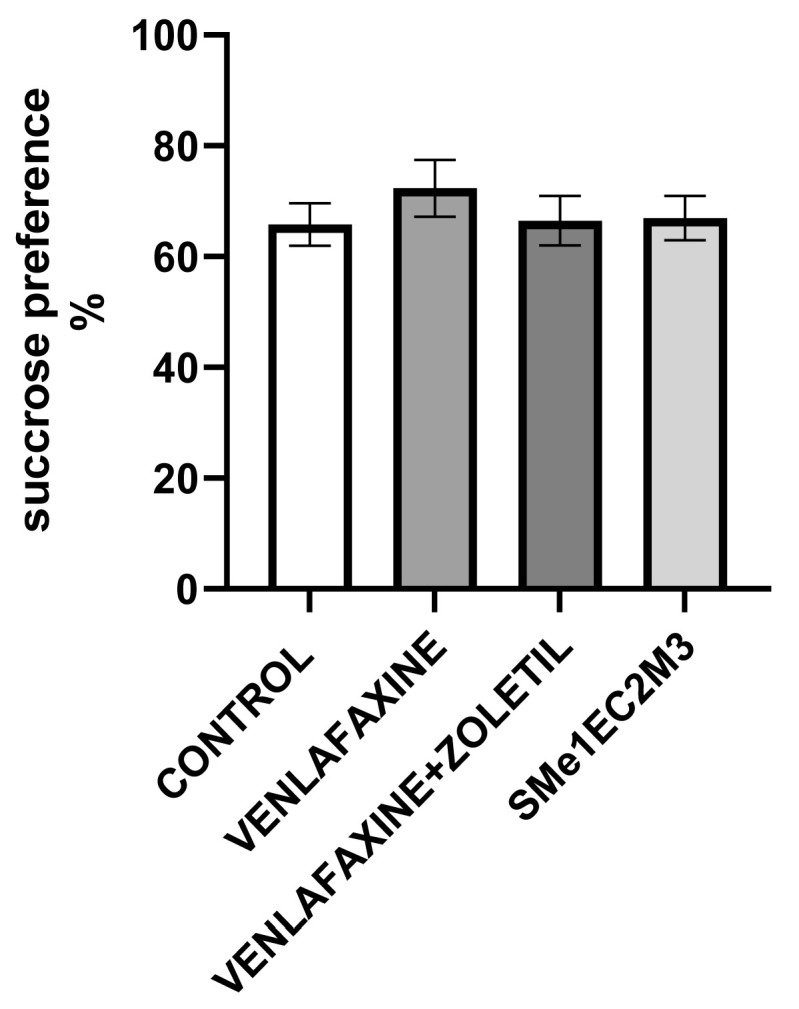
The initial sucrose preference. Means are represented on bars ± SEM (n = 9–10 animals/group). One-way ANOVA did not reveal any significant changes.

We performed a battery of behavioral tests that cover a comprehensive analysis of anhedonia, depressive-like and anxiety-like behavior, spontaneous locomotion, motivation and cognition, particularly recognition memory, to test the antidepressant and anxiolytic efficacy of zoletil + venlafaxine (Alventa 150 mg) and the compound SMe1EC2M3. Before assigning the animals into experimental groups, we performed the sucrose preference test to avoid any significant changes in sucrose consumption between the groups before starting the experiment. We found no statistically significant differences in the experimental groups (Figure 2).

**Figure 3:**
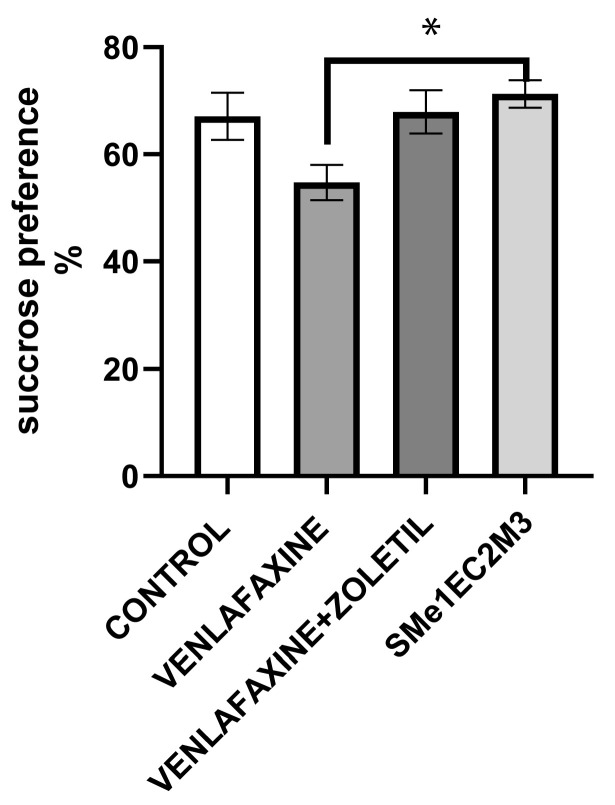
The effect of selected compounds on sucrose preference. Means are represented on bars ± SEM (n = 9–10 animals/group). One-way ANOVA and Tukey’s post hoc test revealed that Sme1Ec2M3 did significantly heighten the sucrose consumption (*p* < 0.05) in comparison to venlafaxine group. Significantly different values are marked with * *p* < 0.05.

To assess anhedonia as one of the main symptom of depression, we used the sucrose preference test. The preference was calculated as a percentage of the volume of sucrose intake over the total volume of the water intake. In the sucrose preference test (SPT), after finishing exposure, one-way ANOVA followed by Tukey’s post hoc test revealed significantly increased sucrose consumption in the Sme1EC2M3 group in comparison with the venlafaxine animals [F(3,33) = 4.028; *p* < 0.05, Figure 3].

**Figure 4:**
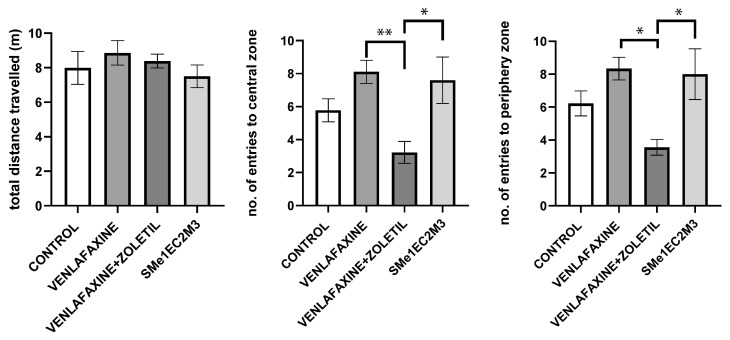
The effect of selected compounds on parameters measured in open field test. Means are represented on bars ± SEM (n = 9–10 animals/group). One-way ANOVA followed by Tukey’s post hoc test revealed that animals exposed to venlafaxine entered the central zone significantly more often (*p* < 0.01) than venlafaxine + zoletil group, as well as SMe1EC2M3-treated animals in comparison with venlafaxine + zoletil group (*p* < 0.05). The number of entries to periphery zone statistics revealed a significant increase in venlafaxine + zoletil group in comparison with both SMe1Ec2M3 group (*p* < 0.05) and venlafaxine (*p* < 0.05) group. Significantly different values are marked with * *p* < 0.05, ** *p* < 0.01.

We used the open field test to observe spontaneous locomotion and anxiety-like behavior based on entrance to the central or periphery zones of the field. In the open field test (OF), the analysis did not reveal any significant differences in the total distance travelled between experimental groups. One-way ANOVA revealed a significant effect of treatment on the number of entries to the central zone [F(3,33) = 5.289; *p* < 0.01]. Tukey’s post hoc analysis revealed that animals exposed to venlafaxine entered the central zone significantly more often (*p* < 0.01) than the venlafaxine + zoletil group, as well as the Sme1EC2M3-treated animals in comparison with the venlafaxine + zoletil group (*p* < 0.05). One-way ANOVA also revealed a significant effect of treatment on the number of entries to the periphery zone [F(3,33) = 4.755; *p* < 0.05]. Tukey’s post hoc test revealed a significant increase in the venlafaxine + zoletil group in comparison with both the SMe1EC2M3 (*p* < 0.05) and venlafaxine (*p* < 0.05) groups (Figure 4).

**Figure 5:**
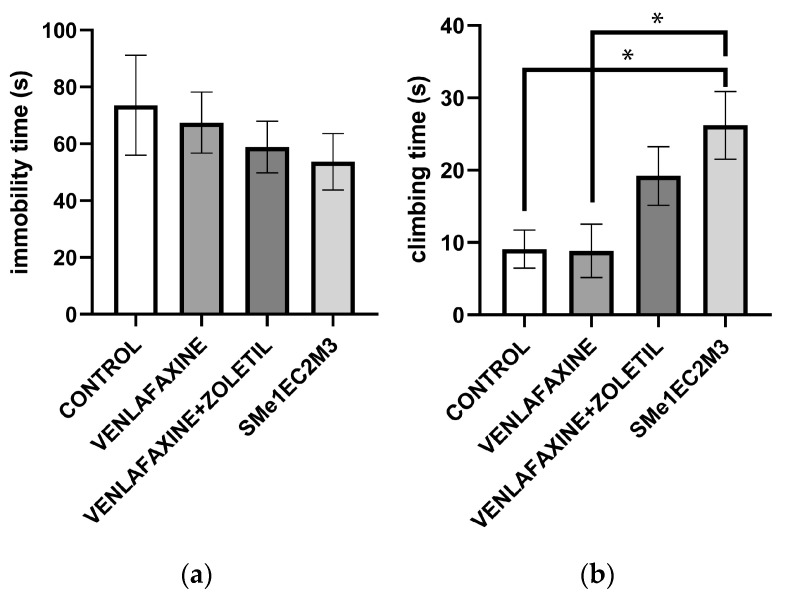
The effect of selected compounds on immobility time (**a**) and climbing time (**b**) in forced swim test. Means are represented on bars ± SEM (n = 9–10 animals/group). Kruskal–Wallis followed by Dunn’s test found significant difference in time spent climbing in SMe1EC2M3 group (*p* < 0.05) in comparison to venlafaxine group. No significant differences were found in time spent in immobility. Significantly different values are marked with * *p* < 0.05.

We also measured depressive-like behavior in the forced swim test (FST). The Kruskal–Wallis followed by Dunn’s test found a significant difference in the time spent climbing in the SMe1EC2M3 group [F(3,33) = 4791; *p* < 0.05] in comparison to both the control (*p* < 0.05) and venlafaxine (*p* < 0.05) groups. No significant differences were found in the time spent in immobility; however, a slightly reduced immobility time was seen in the SMe1EC2M3 group in comparison with the control group (Figure 5).

**Figure 6:**
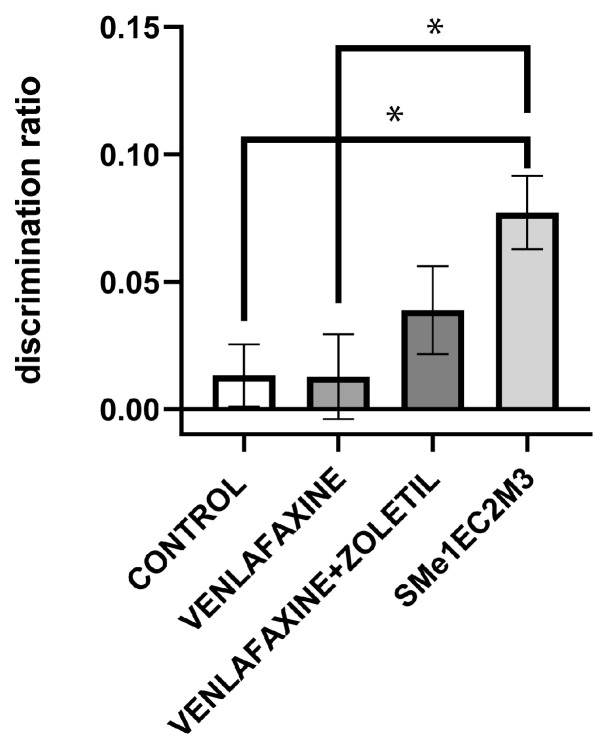
The effect of selected compounds on discrimination ratio in novel object recognition test. Means are represented on bars ± SEM (n = 10 animals/group). One-way ANOVA followed by Tukey’s post hoc test revealed that animals exposed SMe1EC2M3 had significantly heightened discrimination ratio in comparison to control and venlafaxine groups. Significantly different values are marked with * *p* < 0.05.

The novel object recognition test (NOR) is used to assess memory and cognition, particularly recognition memory. The one-way ANOVA followed by Tukey’s post hoc test revealed that the animals exposed to SMe1EC2M3 had a significantly heightened [F(3,33) = 4120; *p* < 0.05] discrimination ratio in comparison to the control (*p* < 0.05) and venlafaxine (*p* < 0.05) groups (Figure 6). The discrimination ratio is defined as the difference in exploration time for the novel object (in sec) divided by the total exploration time (in sec).

**Figure 7:**
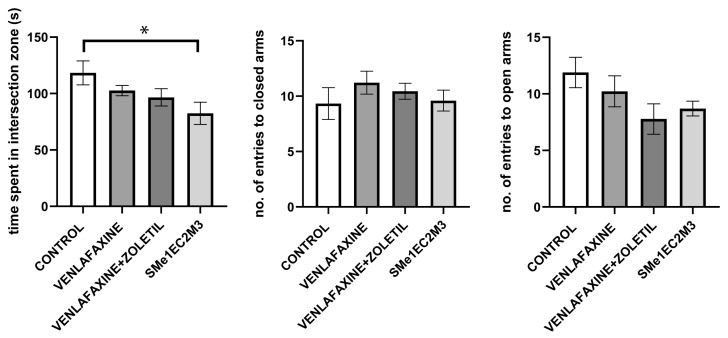
The effect of selected compounds on number of entries to closed and open arms of the maze and time spent in intersection of elevated plus maze. Means are represented on bars ± SEM (n = 9–10 animals/group). One-way ANOVA followed by Tukey’s post hoc test revealed that animals exposed to SMe1EC2M3 spent significantly less time in intersection zone of the elevated plus maze (*p* < 0.05). However, there were no significant changes in number of entries to both closed and open arms of the maze. Significantly different values are marked with * *p* < 0.05.

In the elevated plus maze test (EPM), we assessed the level of anxiety-like behavior by using the indicator of the time the animals spent in the closed and open arms. We did not observe any significant changes in time spent in the open arms and time spent in the closed arms of the maze (Figure 7).

**Figure 8:**
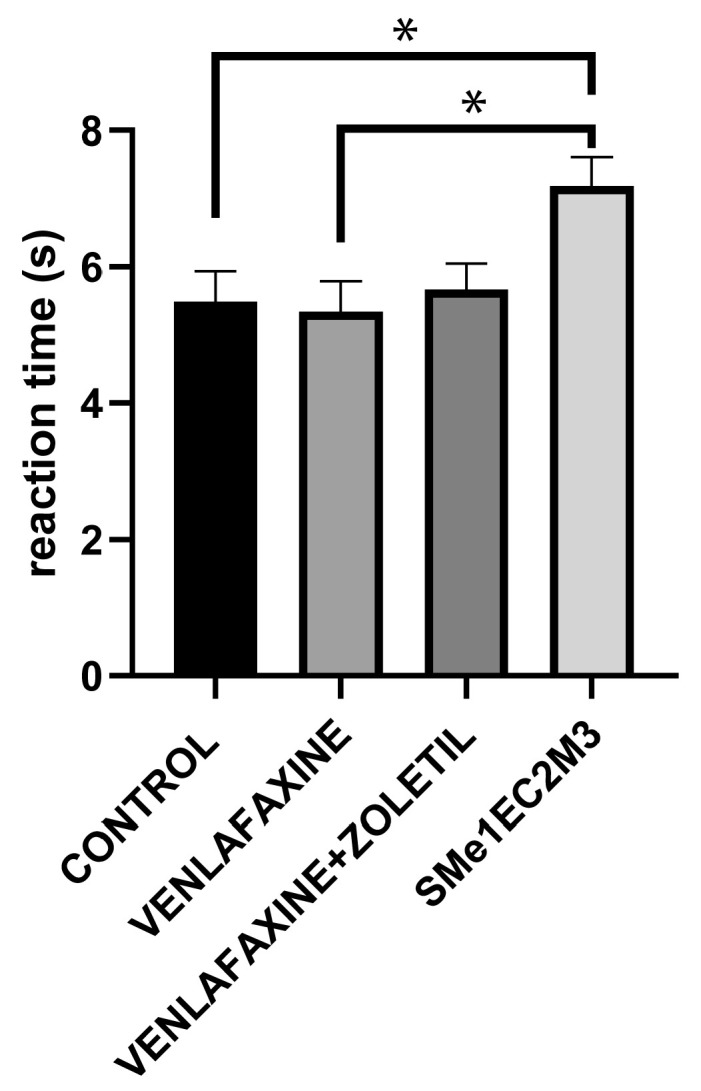
The effect of selected compounds on reaction time in tail flick test. Means are represented on bars ± SEM (n = 9–10 animals/group). One-way ANOVA followed by Tukey’s post hoc test revealed that animals exposed to SMe1EC2M3 had significantly heightened (*p* < 0.05) reaction time in comparison to both control and venlafaxine groups. Significantly different values are marked with * *p* < 0.05.

The tail flick test was used to measure the reaction time to painful stimuli to assess nociception. The one-way ANOVA revealed a significant effect of treatment [F(3,33) = 4.218; *p* < 0.05]. Tukey’s post hoc test revealed a significant increase in reaction time to painful stimuli in the SMe1EC2M3 group (*p* < 0.05) in comparison to both the control and venlafaxine groups (Figure 8).

## 3. Discussion

Our results showed that the negative effects of CMS in WKY rats are partially reduced by the administration of SMe1EC2M3, a newly synthesized pyridoindole derivative. However, a new therapeutic approach combining seven days’ treatment with zoletil followed by venlafaxine administration does not have a significant effect on the amelioration of depression-like behavior in this animal model of TRD.

The forced swim test (FST) is able to differentiate between antidepressants that work through serotonergic mechanisms or noradrenergic mechanisms. Serotonergic antidepressants, including SSRIs, selectively increase swimming behavior while noradrenergic compounds selectively increase the climbing [27]. Further reboxetine, norepinephrine and dopamine reuptake inhibitor (NDRI) is an antidepressant showing rather increased climbing behavior in FST compared to antidepressants such as fluoxetine, which acts mainly on 5-HT receptors and increases the swimming time [28]. Also, chronic 14-day administration with the SSRIs citalopram and desipramine as tricyclic antidepressants reduce immobility time in rats [29]. The chronic administration of SMe1EC2M3 influenced FST parameters, such as by increasing the time spent in active climbing and mildly reducing immobility time. These results correspond with previous findings on the acute administration of SMe1EC2M3 and forced swim test outcomes [19].

Active behavior in the FST could lead to escape; on the other hand, passive behavior may preserve energy while waiting for a possible chance to escape. SSRIs have been shown to postpone the transition from active to passive strategies, while a depressive state contributes to this transition [27].

The results from the tail flick test showed a significant increase in reaction time in the SMe1EC2M3 group, which suggests anti-nociceptive properties of our tested compound. Other studies report that depression highly co-occurs with chronic pain. Patients with chronic pain are at higher risk of depression and the presence of pain in depressed patients had a less favorable outcome for the depression [30]. Preclinical studies showed that antidepressants acting on noradrenergic systems such as venlafaxine also have an anti-nociceptive effect in rodents [31,32]. In neuropathic pain models, TCAs modulate monoaminergic responses at the spinal cord level and have an anti-nociceptive effect in rodents [33]. Moreover, the inhibitory effects of these antidepressants for neuropathic pain manifest more quickly than their antidepressive effects [34]. Also, the triple reuptake inhibitors involving not only the serotonergic and noradrenergic but also the dopaminergic system have been effective in the relief of pain [35].

The inhibition of noradrenaline reuptake increases analgesic effects, predominantly through α2-adrenergic receptors in the dorsal horn of the spinal cord. The α2-adrenergic receptors are coupled to the inhibitory G protein (Gi/o), which inhibits the presynaptic voltage-gated Ca^2+^ channels that inhibit the release of excitatory neurotransmitters from afferent fibers. In meantime, G-protein-coupled K+ channels are opened on the postsynaptic spinal cord dorsal horn cells, the membranes are hyperpolarized and excitability is decreased [36].

In the novel object recognition test, we observed a higher discrimination ratio or preference to explore a new object over the known one in the SMe1EC2M3 group in comparison to both the control and venlafaxine groups. It is generally known that cognitive impairment might occur during stress and depression in both patients and animal models of depression. This test relies on visual recognition memory and is based on rodents’ exploratory behavior and spontaneous preference for novelty in general [37]. Our results are supported by several studies, where the stressed rodents showed a significantly lower discrimination ratio, which was restored after antidepressant administration in both rats and mice [38,39,40] or ketamine administration in Wistar-Kyoto rats [13].

Both the open field test and the elevated plus maze test are widely used to assess general locomotor activity and anxiety-like behavior in animals. In the open field test, we did not observe any significant differences in distance travelled. We observed a significant decrease in the number of entries to the central zone in the venlafaxine + zoletil group in comparison to venlafaxine and SMe1EC2M3. Similarly, the number of entries to the periphery zone was significantly decreased with venlafaxine + zoletil in comparison to venlafaxine and SMe1EC2M3. However, SMe1EC2M3 did not alter the observed parameters in comparison with the control group. In the elevated plus maze test, the treatment did not significantly change the number of entries to either the open or closed arms of the maze. However, SMe1EC2M3 decreased the time spent in the intersection zone of the maze. The results showed no changes in anxiety-like behavior after treatment with SMe1EC2M3 or venlafaxine + zoletil. Preclinical studies show evidence that WKY rats are naturally less active in the open field test in comparison to SHR, Fisher 344 or Lewis rats [41,42,43]. Similarly, in EPM, WKY rats tend to have a rather decreased locomotory behavior and higher reactivity to aversive stimuli (decreased number of entries to open arms of EPM) in comparison to SHR rats [41]. Nosek et al. (2008), in their study with WKY rats, observed an increased time spent in the intersection zone of the EPM. Rats spent significantly more time in this zone than other rat strains. They interpreted these findings as being connected to more indecisive or ambivalent behavior in WKY rats, which can be seen in clinical depression [44]. These finding might suggest that animals after SMe1EC2M3 treatment exhibit less indecisive or ambivalent behavior, the variable present in clinically depressed patients.

The sucrose preference test showed a significantly reduced sucrose intake in the WKY group treated with venlafaxine compared to the SMe1EC2M3 group. It is well known that CMS may reduce the consumption of sucrose or sucrose preference behavior. Moreover, some studies have confirmed the resistance of WKY animals to venlafaxine [13,45], which contributes to our findings. Moreover, the study by Wright et al. (2020) observed a naturally lower intake of sucrose solution in WKY rats [46]. In our study, we observed a slight but not significant increase in sucrose preference with SMe1EC2M3 treatment; we believe that a possibly longer application period and/or an increase in the dose of SMe1EC2M3 could make the antidepressant effect of the drug even greater. Preclinical studies show that food and water deprivation before the SPT can reduce sucrose preference. Such an effect can be explained by the fact that the animals first seek to quench their thirst after the administration of two bottles during the test and prefer water to sucrose, and then they start to prefer sucrose [47,48]. In addition, water deprivation has been shown to increase the preference threshold for the sucrose solution, as water tastes better in thirsty animals [49] and affects the rate of preference development, suggesting that in non-water-deprived animals, the preference develops more quickly [50]. For these reasons, we should review our sucrose preference test protocol.

## 4. Materials and Methods

### 4.1. Animals

The male Wistar-Kyoto rats (initial weight 200–220g, 3 months old) used in this study were acquired from Dept. of Toxicology and Laboratory Animals Breeding, Centre of Experimental Medicine of the Slovak Academy of Sciences (Dobrá Voda, Slovak Republic). All animals were housed under standard laboratory conditions (temperature: 22 ± 2 °C; humidity: 55 ± 10%) with a 12 h light/12 h dark cycle (lights on at 7 a.m.). Pelleted food and tap water were available ad libitum. All performed experiments were conducted in compliance with the Principles of Laboratory Animal Care issued by the Ethical Committee of IEPT, CEM SAS, and the experimental design was approved by the Animal Health and Animal Welfare Division of the State Veterinary and Food Administration of the Slovak Republic (permit number 6021/2022-220). For the behavioral study, 10 animals per group were used (n = 40).

### 4.2. Drugs

Based on our previous results, we chose the substance SMe1EC2M3 in a dose of 10 mg/kg/day, which could offer an effective therapeutic effect [21]. To be sure that animal the model of pharmacoresistant depression is valid, we choose venlafaxine (Alventa 150 mg, Krka, d. d., Novo mesto, Slovenia), which should not have any therapeutic effect in the TRD animal model [13]. We chose a dose of venlafaxine of 10 mg/kg/day based on the available scientific literature [51,52]. As a potential new therapeutic approach, we chose zoletil (10 mg/kg/day) [53] in the first week of the experiment and continued the treatment with venlafaxine (10 mg/kg/day) in the following 3 weeks. In this experimental work, we administered the drugs per os on biscuit in a maximum volume of 1 mL/kg. Aqua pro injectione was used as a vehicle.

### 4.3. Experimental Design and Group Formation

**Figure 9:**
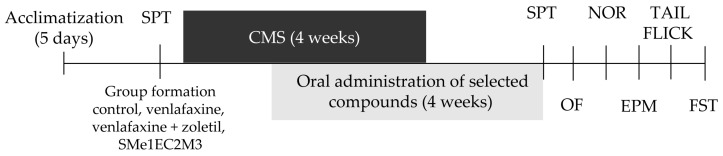
Experimental design. The experimental schedule of applied CMS during the four weeks; the antidepressant treatment with SMe1EC2M3, venlafaxine (Alventa 150 mg) and zoletil in two doses for two consecutive weeks; and the behavioral testing schedule. CMS—chronic mild stress; FST—forced swim test; OF—open field test; SPT—sucrose preference test; NOR—novel object recognition test; EPM—elevated plus maze test.

The rats were acclimatized in the animal housing facility for 1 week prior to the experimental procedures. The animals were divided into 4 experimental groups: stress + vehicle, stress + venlafaxine, stress + SMe1EC2M3 and stress + venlafaxine + zoletil. All experimental animals were exposed to a particular stressor each day. The following types of stressors were used: overcrowding (10 instead of 3 or 4 rats were placed into one T4 cage (590 × 380 × 200 mm of size) for 12 h), air puff (air puffs divided into 45 episodes, each of them lasting 1 min, randomized by a computer for 12 h), wet bedding (1000 mL of water poured into the cage for 12 h), predator stress (5 × 5 × 10 cm large perforated box filled with cat’s litter box content was put into the home cage for 12 h to provide cat odor), food deprivation (food withdrawal for 12 h) and tilted home cage at 45 degree angle for 12 h (Table 1). The animals were exposed to stressors for 4 weeks (Figure 9). Animals were orally treated daily from the 14th day of the CMS for 4 weeks except the venlafaxine + zoletil group, where animals received zoletil in the first week followed by 3-week administration of venlafaxine. The volume was 1 mL/kg body weight. During the experiment, the animals were handled and weighed regularly. The tested compound SMe1EC2M3 was synthesized at the Faculty of Natural Sciences, Comenius University in Bratislava, Slovakia, as a hydrochloride salt. The purity of the compound was greater than 95% as determined by ^1^H-NMR analysis.

### 4.4. Behavioral Analysis

All behavioral tests and the order of the tests were chosen according to our previous studies [19,21,54]. The recovery time for animals between the behavioral tests was set to at least 24 h. The order of the tests was the following: sucrose preference test for assessment of anhedonia followed by open field test, novel object recognition test and elevated plus maze test. These tests were conducted in a behavioral testing room, where the animals were acclimatized for 30 min before each test session. The forced swim test for the assessment of depressive-like behavior was the last test as it is the most stressful event from all chosen behavioral tests. This particular order of behavioral tests should ensure that there were no interactions of the test and the animals’ behavior. All behavioral tests were conducted between 8 a.m. and 1 p.m. during the light phase of the day in a controlled illuminated room with 200 lux. All mazes were cleaned immediately after each individual test with 60% ethanol to remove odor cues. Tested substances were applied after behavioral test. For all tests, the movements of the rats were tracked with a digital camera and analyzed by computer software ANYMAZE™ 7.20 (Stoelting Europe, Dublin, Ireland).

#### 4.4.1. Sucrose Preference Test (SPT)

The sucrose preference test was used to detect anhedonia as a main symptom of MDD in rats. All animals were habituated to the presence of 1% sucrose solution for 24 h. On the 2nd day, we offered them two drinking bottles (one containing 1% sucrose and the other one tap water) for 24 h in their home cages. The position of the two bottles was switched every 2 h within this 24 h to reduce any confounding produced by a side bias. On the 3rd day, the animals’ food and sucrose solution access was removed for 24 h and no tap water intake was allowed for 12 h before starting the test. Following this acclimation and food/water deprivation, the animals were, on the experimental day, housed individually and had a free choice to either drink 1% sucrose solution or regular water for 2 h (8 a.m.–10 a.m.). Sucrose preference was calculated as a percentage of the volume of sucrose intake over the total volume of the water intake.

#### 4.4.2. Forced Swim Test (FST)

For the forced swim test, the rats were placed in a glass cylinder (45 cm tall and 25 cm in diameter) filled with water (24 ± 1 °C) for a 15 min pre-test period to induce depression-like behavior. The following experimental 5 min test started 24 h later. The depth of the water (30 cm) was sufficient to ensure that the animals could not touch the bottom of the container with their hind paws. All animals were returned to their home cage after resting under a heating lamp until dry. The times of immobility, climbing and swimming of each animal were scored manually. Immobility (passive behavior) was defined as immobility without any movements other than those necessary to balance the body and keep the head above the water [24]. Escape-directed (active) behavior were scored separately as vertical movement of the forepaws (climbing).

#### 4.4.3. Open Field (OF)

The open field test consisted of a dark polyvinyl plastic arena measuring 60 × 60 cm surrounded by 25 cm high walls. Each session started by placing the rat in the center of the maze and letting it freely explore the new environment for 5 min. Selected parameters were determined: total distance travelled and number of entries to central and periphery zone.

#### 4.4.4. Elevated plus Maze Test (EPM)

The elevated plus maze test is routinely used for assessing anxiety-like behavior. All parts of the apparatus were made of dark polyvinyl plastic. The open and closed arms of the maze were 50 cm above the floor, 50 cm long and 10 cm wide. Each session lasted 5 min and started by placing the rat in the central intersection area facing the open arm of the maze. Selected parameters were determined: number of entries to open and closed arms of the maze and time spent in intersection zone.

#### 4.4.5. Novel Object Recognition Test (NOR)

The novel object recognition test occurred in a dark polyvinyl plastic arena measuring 60 × 60 cm surrounded by 25 cm high walls. The test is divided into three sessions. The first day starts with habituation to the arena for 5 min. On the second day, we place objects (1 group round object, 1 group pointy objects) in the arena and let animals remember these objects. On the third day, the experiment takes place. The animals are put back into the arena for 3 min, but now in one of the corners there is a known object and on the other there is a novel object. Discrimination ratio was used to determine the ability to remember and recognize the new object. The discrimination ratio is defined as the difference in exploration time for the novel object (in sec) divided by the total exploration time (in sec).

#### 4.4.6. Tail Flick Test

The tail flick test is widely used to assess pain sensitivity (nociception) in rodents. The time it takes for an animal to remove the tail from an intense infrared heat source is used as an index of peripheral pain response. The animal’s tail is placed over a heat source controlled by the experimenter, and a timer automatically begins. When the animal feels pain it immediately flicks its tail away. A sensor detects this movement and stops the timer and switches off the infrared heat source. Reaction time or latency to withdrawal the tail is the primary measure of this reflexive assay. The tail flick apparatus was purchased from Ugo Basile (model 37360) with irradiation set on 20. The irradiation was chosen based on the fact that most of the animals should flick the tail after 4 s of duration of the test [55].

### 4.5. Statistical Analyses

Data analyses were performed using GraphPad Prism 8.3 software. Behavioral data are represented by mean ± SEM. All data were tested for normal distribution using Kolmogorov–Smirnov test. If data were normally distributed, they were analyzed by a one-way analysis of variance (ANOVA) with treatment as main factor followed by Tukey’s post hoc test, if applicable. Data which did not pass the normality test were analyzed by the one-way non-parametric Kruskal–Wallis test followed by Dunn’s multiple comparison test. A value of *p* < 0.05 was considered statistically significant. The outliers were excluded if the data points ranged more than 1.5 interquartile below the first quartile or above the third quartile.

## 5. Conclusions

In conclusion, our results show that treatment with SMe1EC2M3 can be effective in ameliorating some of the depression-like symptoms present in stressed Wistar-Kyoto rats, as an animal model of TRD. Overall, SMe1EC2M3 might represents a potential drug candidate with positive neurobehavioral effects in therapy for pharmacoresistant depression. Also, from the tail flick test and forced swim test, we might assume that SMe1EC2M3 might be predominantly active on noradrenergic systems rather than serotonergic or dopaminergic ones. However, the chronic administration of SMe1EC2M3 and its positive effects in the treatment of TRD should be explored more in depth to obtain a complex overview.

### Limitations of the Study

Depression is more prevalent in females and there is sexual dimorphism not only in the neurobiology of depression but also in the treatment response. Thus, the use of only males could be potential limitation of this study.

SMe1EC2M3 is a potential new antidepressant with a lot of beneficial effects; however, we did not observe reversion in some parameters evaluated, which could be affected by the chosen dose. In this sense, the use of only one dose might also be a limitation.

## Figures and Tables

**Table 1:** The schedule of applied stressors during 4-week CMS procedure.

Stressor	Days of CMS
overcrowding	6, 10, 16, 27
air puff	4, 8, 19, 24
wet bedding	2, 9, 20, 23
tilted cage	1, 11, 18, 25
predator stress	5, 13, 17, 26
food deprivation	3, 12, 15, 22

## Data Availability

The data presented in this study are available on request from the corresponding author. The data are not publicly available due to ethical, legal, or commercial reasons.
